# Tumor endothelial marker 1 is upregulated in heart after cardiac injury and participates in cardiac remodeling

**DOI:** 10.1038/s41598-022-14567-2

**Published:** 2022-06-22

**Authors:** Po-Sheng Chen, Wen-Han Feng, Tzu-Hsien Tsai, Yi-Kai Hong, An-Sheng Lee, Kuan-Cheng Chang, Hsing-Chun Chung, Yen-Wen Liu, Chih-Cheng Hsieh, Yi-Hsian Fang, Pei-Jung Yang, Chawn-Yau Luo, Ping-Yen Liu, Tsung-Lin Cheng, Yi-Heng Li

**Affiliations:** 1 Department of Internal Medicine, National Cheng Kung University Hospital, College of Medicine, National Cheng Kung University, 138 Sheng Li Road, Tainan, 704 Taiwan; 2 Institute of Clinical Medicine, National Cheng Kung University Hospital, College of Medicine, National Cheng Kung University, 138 Sheng Li Road, Tainan, 704 Taiwan; 3 Department of Internal Medicine, Kaohsiung Municipal Ta-Tung Hospital, Kaohsiung Medical University Hospital, Kaohsiung Medical University, Kaohsiung, Taiwan; 4 Department of Internal Medicine, Kaohsiung Medical University Hospital, Kaohsiung Medical University, Kaohsiung, Taiwan; 5 Department of Internal Medicine, Ditmanson Medical Foundation Chia-Yi Christian Hospital, Chiayi City, Taiwan; 6 Department of Dermatology, National Cheng Kung University Hospital, College of Medicine, National Cheng Kung University, Tainan, Taiwan; 7 International Center of Wound Repair and Regeneration, College of Medicine, National Cheng Kung University, Tainan, Taiwan; 8 Department of Medicine, MacKay Medical College, New Taipei City, Taiwan; 9 Department of Internal Medicine, China Medical University Hospital, Taichung, Taiwan; 10 School of Medicine, China Medical University, Taichung, Taiwan; 11 Department of Surgery, National Cheng Kung University Hospital, College of Medicine, National Cheng Kung University, Tainan, Taiwan; 12 Orthopedic Research Center, Kaohsiung Medical University, Kaohsiung, Taiwan; 13 Regenerative Medicine and Cell Therapy Research Center, Kaohsiung Medical University, Kaohsiung, Taiwan; 14 Department of Physiology, School of Medicine, College of Medicine, Kaohsiung Medical University, Kaohsiung, Taiwan

**Keywords:** Cell biology, Cardiology

## Abstract

Tumor endothelial marker 1 (TEM1) is a transmembrane glycoprotein that appears on mesenchymal lineage-derived cells during embryogenesis, but its expression greatly reduces after birth. Re-upregulation of TEM1 is found in tumor angiogenesis, organ fibrosis and wound healing indicating its potential role in tissue remodeling and repair. The expression level and function of TEM1 in adult heart are unknown. In explanted hearts from heart failure (HF) patients received cardiac transplantation, immunofluorescence staining showed TEM1 was expressed in cardiomyocytes (CMs) and cardiac fibroblasts. Bioinformatics analysis showed TEM1 upregulation in mouse heart after coronary ligation. Cardiac TEM1 expression was reconfirmed in mouse HF induced by coronary ligation or doxorubicin injection. TEM1 expression increased in cultured CMs stimulated with mechanical stretch, doxorubicin and hypoxia. Further studies showed recombinant TEM1 (rTEM1) was a functional protein that influenced cell behaviors of CMs. It directly activated Erk and Akt through interaction with PDGF receptor. TEM1^lacZ/lacZ^ mice had less collagen deposition and worse cardiac function than wild type mice. These results indicate that TEM1 expression increases in the heart after cardiac injury and works as a functional protein that participates in cardiac remodeling.

## Introduction

Tumor endothelial marker 1 (TEM1), also known as endosialin or CD248, is a highly glycosylated transmembrane protein which belongs to a C-type lectin domain group 14 protein family^1,2^. TEM1 has 757 amino acids and its protein structure is composed of six domains, including an extracellular N-terminal C-type lectin domain (D1), a sushi domain (D2), an epidermal growth factor (EGF) domain (D3), a mucin-like region (D4), a transmembrane domain (D5), and a cytoplasmic tail (D6)^3,4^. The other proteins in the family, including thrombomodulin, CLEC14A and CD93, share a similar molecular structure and contain C-type lectin domain^1,2^. Although in the same protein family, TEM1 has a distinctive expression pattern that restricts to mesenchymal lineage-derived cells only during embryogenesis. Its expression dramatically reduced after birth with minimal or undetectable levels in normal adult tissues^1,5^. In vitro study showed that TEM1 re-expression could be induced by hypoxia in fibroblast and glioblastoma cell lines, mainly through the activation of hypoxia inducible factor-2^6^. TEM1 is upregulated during organ fibrosis, atherosclerosis, and wound healing^7–12^. Expression of TEM1 in these pathological conditions and its exclusive expression on mesenchymal/stromal cells suggest that TEM1 plays potential roles in tissue remodeling and repair after damage.

Heart failure is the final common pathway after cardiac injury. Complex mechanisms composing of interactions of diverse immune cells, inflammatory cytokines and adaption of cardiac cells, especially hypertrophy, hyperplasia and death of cardiomyocytes, are responsible for the remodeling process of heart failure^13,14^. Cardiomyocytes occupy 85% of the mass in adult mammalian heart and follow by cardiac fibroblasts, endothelial cells, and perivascular cells^15^. In response to cardiac injury, cardiomyocytes to cardiomyocytes and cardiomyocytes to interspersed interstitial cardiac fibroblasts can interact each other via the release of paracrine or autocrine factors^16^. In normal mouse, TEM1 expression in heart could be detected during the early stage of embryonic development and there is only minimal TEM1 expression in the heart immediately after birth^5^. Although the protein level of TEM1 is limited, its gene expression can be detected in normal adult murine and human tissues, including the cardiovascular system^5,17^. The spatial expression pattern and potential physiological function of TEM1 in heart after injury have never been investigated. The major purpose of this study is to explore the change of cardiac TEM1 expression after injury and the influences of TEM1 on biological functions of cardiomyocytes during cardiac remodeling.

## Materials and methods

### TEM1 in human heart failure

Human cardiac specimens from left ventricle were collected from the explanted hearts of 2 patients (both were male, patient A was 61 years old and patient B was 57 years old) with terminal heart failure undergoing cardiac transplantation in the National Cheng Kung University Hospital, Tainan, Taiwan. Normal human left ventricle tissues purchased from US Biomax, Inc., Derwood, USA (category number: HuFPT056) were used as control. All studies were carried out in accordance with the World Medical Association Declaration of Helsinki and the study protocols were approved by the Institutional Review Board (IRB) of the National Cheng Kung University Hospital, Tainan, Taiwan (IRB number B-ER-106-116).

### TEM1 in mouse heart failure induced by ischemia

Heart failure induced by ischemia was produced by coronary ligation. The mice (male C57BL/6 mice, 8–12 weeks) were anesthetized, intubated and connected to a volume-cycled rodent ventilator (Minivent Mouse Ventilator, type 845, Germany). Median sternotomy was performed and the left anterior descending (LAD) artery was suture ligated to produce permanent myocardial infarction (MI).

### TEM1 in mouse heart failure induced by cytotoxicity

Heart failure induced by cytotoxicity was produced by doxorubicin injection. The mice (male C57BL/6 mice, 8–12 weeks) received intraperitoneal injection of doxorubicin 5 mg/kg/week for 4 weeks to an accumulated dosage of 20 mg/kg. All animal experiments were performed in the National Cheng Kung University Hospital, Tainan, Taiwan with the approval of institutional animal care and use committee (NCKUH number 109056). All methods were performed in accordance with the guidelines of International Council for Laboratory Animal Science and the results were reported in accordance with ARRIVE guidelines.

### Bioinformatics analysis

The mice hearts between mid-LAD artery and apex were obtained at day 5 after coronary ligation (n = 3) and sham operation (n = 3). Expression profiles of RNAs were performed with the next generation sequence (NGS). Total RNA from mice hearts were extracted and the sequencing analysis was performed by Welgene Biotechnology (Taipei, Taiwan). The Agilent's SureSelect Strand-Specific RNA Library Preparation Kit was used for library construction. Differential expression analysis was performed based on cuffdiff (@cufflinks 2.2.1) and Welgene in-house programs. The HISAT2 is a fast and sensitive alignment program based on mapping NGS reads to the genomes^18^. The NGS and bioinformatics analysis were performed to search genes with high expression levels by using the Database for Annotation, Visualization and Integrated Discovery (DAVID) v6.8.1.

### Immunohistochemistry and immunofluorescence staining

The paraffin-embedded sections of heart were incubated with primary antibody for TEM1 (Proteintech). We used DAKO EnVision System, HRP kit that consists of secondary antibodies covalently linked to peroxidase-coated polymer conjugate for immunoperoxidase staining. Diaminobenzidine chromogen was used as color substrate for visualization. For immunofluorescence staining, the primary antibodies for troponin (cardiomyocyte marker, 1:2000 Abcam), fibroblast activation protein (FAP, cardiac fibroblast marker, 1:1000 Abcam) and TEM1 (1:1000, ATLAS) were used. Secondary antibodies are goat anti-mouse IgG Alexa Fluor 546 and goat anti-rabbit IgG Alexa Fluor 488. Nuclei were counterstained with 4′,6-diamidino-2-phenylindole (DAPI, Sigma). The specimens were evaluated by Nikon TE2000 confocal microscope.

### TEM1-deficient mice

TEM1-deficient mice (TEM1^lacZ/lacZ^ mice), in which TEM1 exon was disrupted and replaced by lacZ gene driven by TEM1 promoter, were bred in our laboratory as previously described^5^. The product of LacZ gene, beta-galactosidase, in tissue was detected by staining with Xgal (5-bromo-4-chloro-3-indolyl-b-galactopyranoside) which resulted in deep blue color.

### Cell culture

Rat cardiomyoblast cell line (H9C2) was purchased from the Food Industry Research and Development Institute, Hsin Chu, Taiwan. Human cardiac fibroblast cell line was purchased from Cell Applications (San Diego, CA). Cells were maintained in Dulbecco’s modified Eagle’s medium and 10% fetal bovine serum (FBS) containing 1% penicillin/streptomycin in humidified atmosphere with 5% CO_2_ at 37 °C.

### Stress experiments

We used mechanical stretch, doxorubicin or hypoxia, to stress the H9C2 cells and observe TEM1 expression. In mechanical stretch study, the cells were subjected to cyclic stretch with 10% elongation at a frequency of 60 cycles/min for 6 h using ST-190-XY cell stretch system. In doxorubicin study, the cells were treated with 1 µM doxorubicin for 24 h. In hypoxia study, the cells were placed in a hypoxic incubator prefilled with only 1% oxygen for 24 h. Western blot was used to detect TEM1 expression of cells at different time points during stress.

### Western blot

Cells were homogenized and separated using SDS-PAGE in phosphate-buffered saline (PBS). Samples were transferred to polyvinylidene difluoride (PVDF)-plus membranes and incubated with appropriate antibodies. The blot membranes were cut before hybridization with antibodies. A chemiluminescence reagent (PerkinElmer) was used to detect the signal and band intensity was quantified using AlphaImager 2200 software (Alpha Innotech, San Leandro, CA).

### Recombinant TEM1 (rTEM1)

Recombinant extracellular domains (D1-4) of TEM1 protein were produced in our laboratory. In brief, the pSecTag2-A vector containing human TEM1 with amino acid residues 1–639 was constructed. This vector was transfected into HEK293 cell line, and rTEM1 protein was induced by insulin and purified from the conditioned medium using nickel-chelating sepharose.

### Cell proliferation

We used 5-bromo-2-deoxyuridine (BrdU) assay to evaluate cell proliferation. After receiving indicated treatment for cells, 10 mM BrdU was added and followed by incubation for 2 h. The nuclear incorporation of BrdU was measured using a cell proliferation ELISA kit (Roche Diagnostics, Mannheim, Germany).

### Cell hypertrophy

Cells were fixed after receiving indicated treatment. Phalloidin-conjugated Alexa Fluor 546 (A22283, Invitrogen) was used for actin staining and DAPI for nuclei staining. The images were acquired by Olympus DP72 microscopy. The cell outline was traced and measured using software (MetaMorph, Molecular Devices, Sunnyvale, CA). At least 10 cells were measured for cell area and averaged for each treatment.

### Cell apoptosis

Cell apoptosis was induced by adding doxorubicin (1 µM). The severity of apoptosis was measured by quantification of DNA fragments using a cell death ELISA kit (Roche Diagnostics, Mannheim, Germany).

### Actin filament alignment

The analysis of actin alignment is processed using Fiji/ImageJ software (NIH, USA) as previously described^19^. After staining actin with phalloidin-conjugated Alexa Fluor 546 (A22283, Invitrogen), the acquired immunofluorescence images were converted to 16-bit gray-scale images. Fast Fourier transform analysis for intracellular actin alignment was measured using Directionality plug-in for Fiji. Thus, a normalized histogram in which the amount of actin presented between 0° and 180° was generated. The sum of distribution of actin in all directions is 1 in the normalized histogram. The standard deviation (SD) of actin distribution between each degree was calculated. A higher SD indicates a more uniform orientation of actin than a lower one.

### Cell contractility

Contractile cardiomyocytes were derived from human embryonic stem cells (hESCs) (RUES2 cells, a gift from Dr. Hsieh PCH and Lu J from the Academia Sinica, Taipei, Taiwan). A serum-free, monolayer direct cardiac differentiation protocol by treating hESCs sequentially with the small molecules Chir99021 (4423, Tocris Bioscience) and IWR-1 (I0161, Sigma-Aldrich) was used as previously described^20^. The hESC-derived contractile cells were treated with doxorubicin (5 µg/mL) for 48 h. Then, rTEM1 (100 nM) or saline was added and incubated for another 48 h. A camera was mounted on an inverted microscope to acquire the images of cardiomyocytes contraction for offline analysis. The MUSCLEMOTION software was used to measure cell contraction amplitude and velocity^21^.

### Echocardiography

The mice were anesthetized with 1.5% isoflurane mixed with oxygen. The left ventricular (LV) end-systolic, end-diastolic dimension and ejection fraction (EF) were measured in standard views using a 12 MHz phased array transducer (Sonos 5500; Hewlett-Packard Inc, Andover, MA). The LVEF and mass were calculated according to Teichholz methods.

### Pressure–volume loop analysis

Pressure–volume loop analysis was performed in mice according to the method described previously^22^. A microtip 1.4 F catheter (SPR-839, Millar Instruments, Houston, TX) was inserted into the right carotid artery of mice. The arterial pressure was recorded and the catheter was advanced to LV guided by pressure tracing. The signals of pressure and volume were continually recorded by using a conductance system (MPVS Ultra, emka TECHNOLOGIES, Paris, France) coupled to a digital converter (ML-870, AD Instruments, Colorado Springs, CO). LVEF was derived from the pressure–volume diagram.

### Coimmunoprecipitation

After treatment with 1 μM rTEM1 for 1 h at 37 °C, H9C2 cells were lysed with lysis buffer. After centrifugation, the supernatants were preclarified with protein A/G-Sepharose beads (BioVision) and then subjected to immunoprecipitation using 1 μg anti-TEM1 antibody (Proteintech) or rabbit IgG (Arigo) for overnight at 4 °C. The immunoprecipitates were washed, suspended in sample buffer and subjected to western blot with anti-PDGFR-β (Cell signaling) and anti-TEM1 antibodies.

### Statistical analysis

Data were presented as mean ± standard error (SE). Comparisons between two groups were made by Mann Whitney U test. For multiple comparisons of groups, one-way ANOVA was used and followed by Bonferroni post hoc analysis. All statistical analyses were performed using SPSS 22 (SPSS Inc. Chicago, IL, USA). A *p* value < 0.05 is considered to be statistically significant.

## Results

### TEM1 in human heart failure

**Figure 1:**
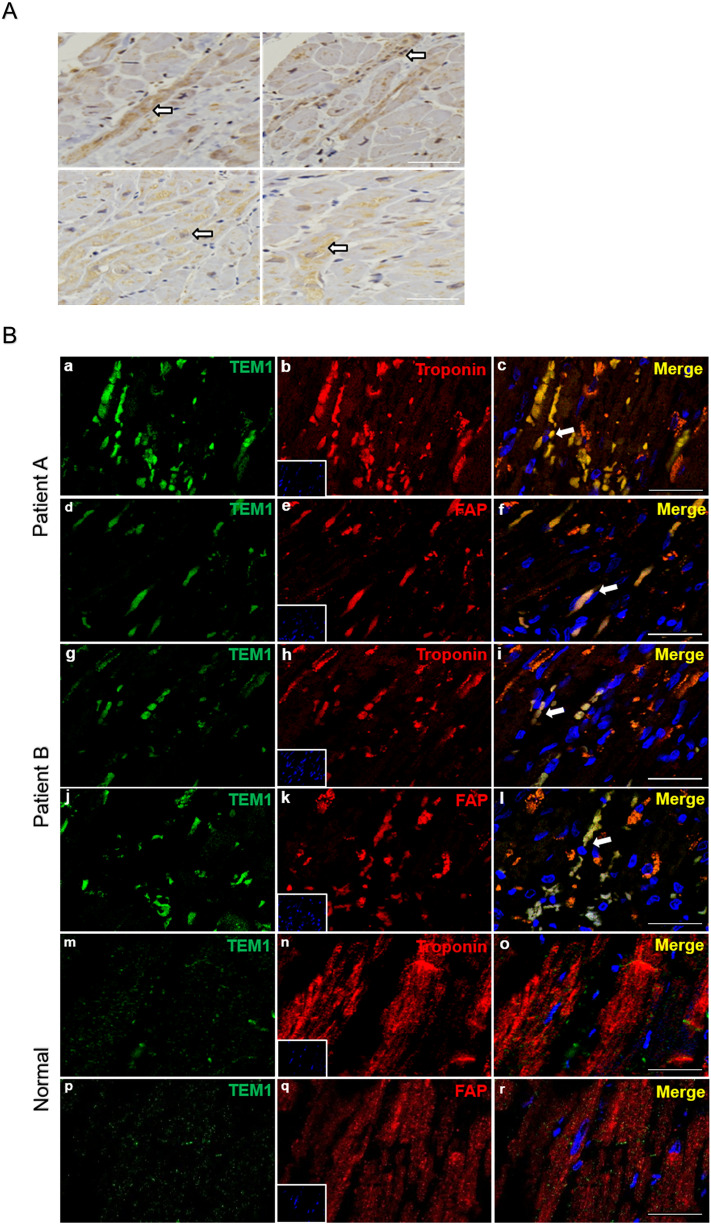
TEM1 in human heart failure. (**A**) Immunohistochemical staining of TEM1 in a cardiac specimen of left ventricle from an explanted heart of patient A received cardiac transplantation. The cell numbers with positive staining in the 4 cross sections were counted and averaged. The ratio of positive staining cells/total cells is 0.62 ± 0.03. Arrows indicate TEM1 staining (brown) in cells. Scale bar = 100 μm. (**B**) Immunofluorescence staining of TEM1 in cardiac specimens of left ventricles from 2 explanted hearts of patient A and B received cardiac transplantation. Representative images show immunostaining for TEM1 (green), troponin (red, cardiomyocyte marker) and fibroblast-activation protein (red, FAP, cardiac fibroblast marker). Nucleus was stained with DAPI (blue). The cell numbers with positive staining in 3 cross sections were counted and averaged in each patient. The ratio of positive staining cells/total cells is: patient A, troponin + TEM1: 0.40 ± 0.02, FAP + TEM1: 0.19 ± 0.02. patient B, troponin + TEM1: 0.34 ± 0.02, FAP + TEM1: 0.29 ± 0.03. Arrows indicate the TEM1 staining (merge) in cells. Scale bar = 25 μm.

Cardiac specimens were taken from 2 explanted hearts of patients with terminal heart failure. The immunohistochemical study of patient A found TEM1 was abundantly expressed in human myocardium (Fig. 1A). To further identify the cell types that express TEM1 in the heart, double immunofluorescence staining of patient A and B showed TEM1 expressed in cardiomyocytes and some of them appeared in cardiac fibroblasts (Fig. 1B). There was minimal TEM1 expression in normal human left ventricular tissue.

### TEM1 in mouse heart failure

**Figure 2:**
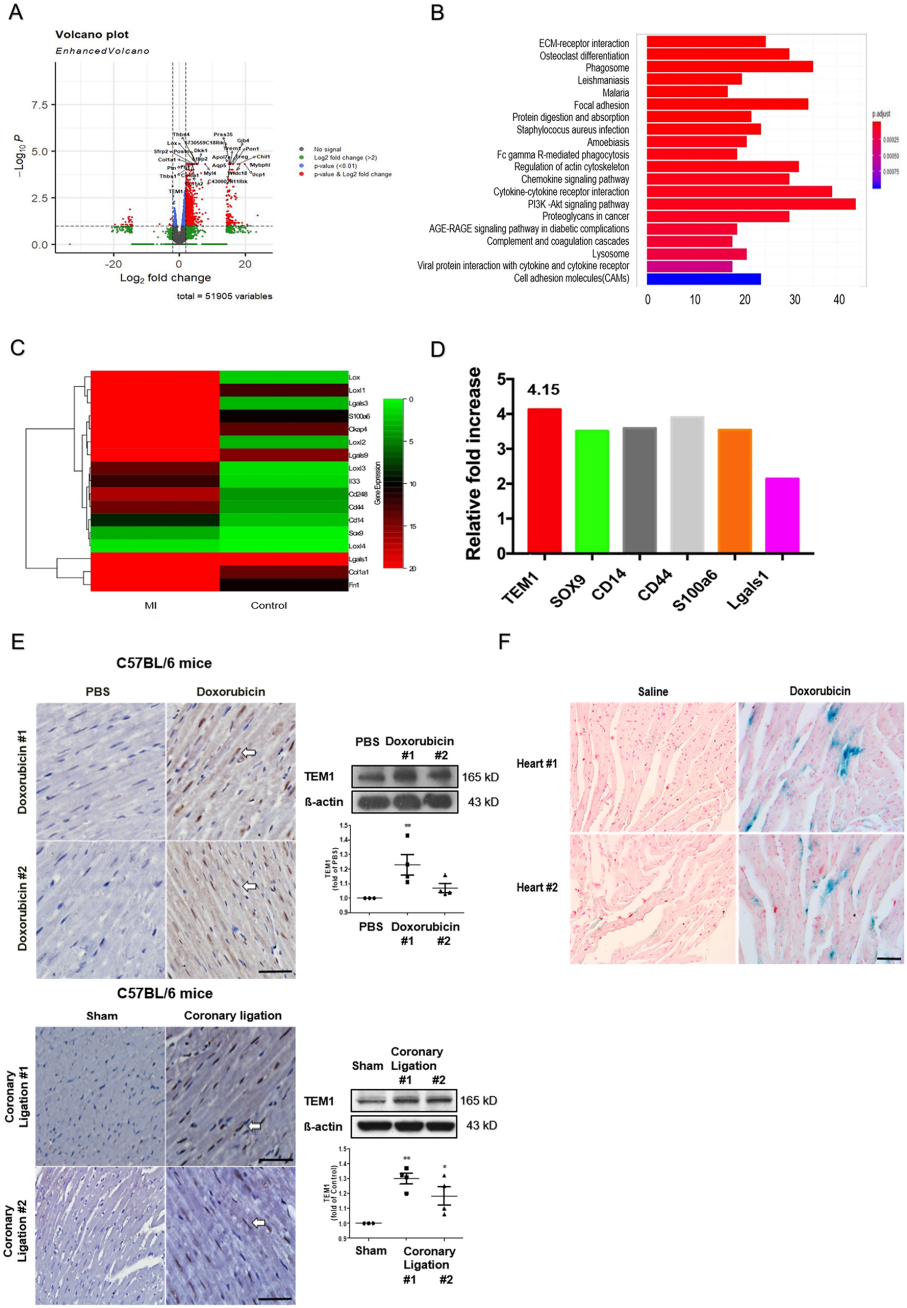
TEM1 in mouse heart failure. (**A**) The pattern of differential expression genes between post-MI and control mice from deep RNA sequencing by volcano plot. (**B**) Canonical pathways significantly associated with the differentially expressed genes in post-MI compared to control mice. The X-axis indicates gene count and Y-axis indicates different pathways. The column color reflects *p* value: red represents the smallest value; blue represents the biggest value. The gradual color ranged from red to blue represents the changing process of *p* value from small to big value. The z-score represents the magnitude of significant activation. (**C**) The heatmap reveals relative expression abundance of differential expressed genes from post-MI and control mice with Z-score values. (**D**) The expression of selected mRNA involved in extracellular matrix-receptor interaction was quantified by PCR array in post-MI and control mice. The relative expression of TEM1 was upregulated to 4.15 times in post-MI mice compared to control mice. MI, myocardial infarction; PCR, polymerase chain reaction. (**E**) Immunohistochemical staining and western blotting of TEM1 in cardiac specimens of the 2 mice (#1 and #2) received doxorubicin injection (upper panel) or 2 mice (#1 and #2) received coronary ligation (lower panel). Control group included 2 mice received PBS injection and 2 mice received sham operation. Arrows indicate TEM1 expression in cells. Scale bar = 100 μm. **p < 0.01 and ***p < 0.001 compared to control group with saline injection or sham operation. N = 3 or 4 for the western blotting. Original images of western blot were provided in “Supplemental file”. (**F**) TEM1-lacZ activity was detected by Xgal staining (deep blue color) in 2 hearts (#1 and #2) of TEM1^LacZ/LacZ^ mice after saline or doxorubicin injection. Scale bar = 25 μm.

Bioinformatics analysis demonstrated that there were a total of 1062 up-regulated genes that showed twofold changes in post-MI mice compared to the sham group (Fig. 2A). The extracellular matrix (ECM)-receptor interaction is one of the top 20 canonical pathways according to the Gene ontology biologic process analysis and Kyoto Encyclopedia of Genes and Genomes (KEGG) pathway (Fig. 2B). The heat map of the significant twofold changed genes about ECM-receptor interaction was shown in Fig. 2C. Interestingly, TEM1 gene expression in the heart was up-regulated about 4.15 times in post-MI mice compared to sham group (Fig. 2D). Coronary ligation was performed in 2 mice and doxorubicin injection was also performed in another 2 mice to induced heart failure. Immunohistochemical study and western blot of mouse #1 and #2 showed increased expression of TEM1 after coronary ligation or doxorubicin injection (Fig. 2E). In TEM1^lacZ/lacZ^ mice, TEM1-lacZ activity in heart was detected by X-gal staining. Doxorubicin treatment induced the expression of beta-galactosidase activity in myocardium of 2 TEM1^lacZ/lacZ^ mice indicating TEM1 gene was upregulated after cardiac injury (Fig. 2F).

### TEM1 in stressed cardiomyocytes

**Figure 3:**
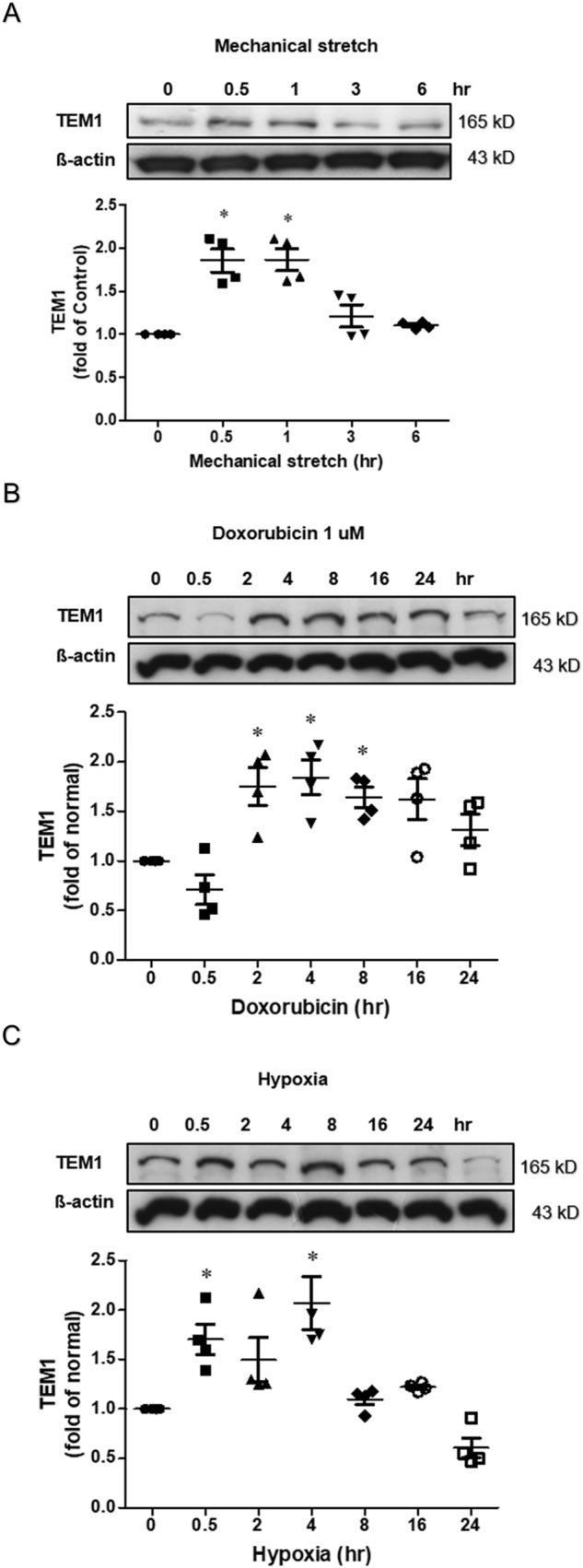
TEM1 in cultured cardiomyocytes. (**A**) H9C2 cells were subjected to mechanical stretch for 6 h. (**B**) H9C2 cells received doxorubicin (1 µM) treatment for 24 h. (**C**) H9C2 cells were exposed to hypoxia incubator prefilled with 1% oxygen for 24 h. The expression levels were analyzed by western blot and expressed as a ratio of baseline before stress (n = 4). *p < 0.05, **p < 0.01 and ***p < 0.001 compared to normal without stress. Original images of western blot were provided in “Supplemental file”.

Stresses on cultured cardiomyocytes (H9C2 cells), including mechanical stretch (Fig. 3A), doxorubicin treatment (Fig. 3B) and hypoxia stimulation (Fig. 3C), promoted an increase of TEM1 expression.

### Influences of rTEM1 on cell behaviors

**Figure 4:**
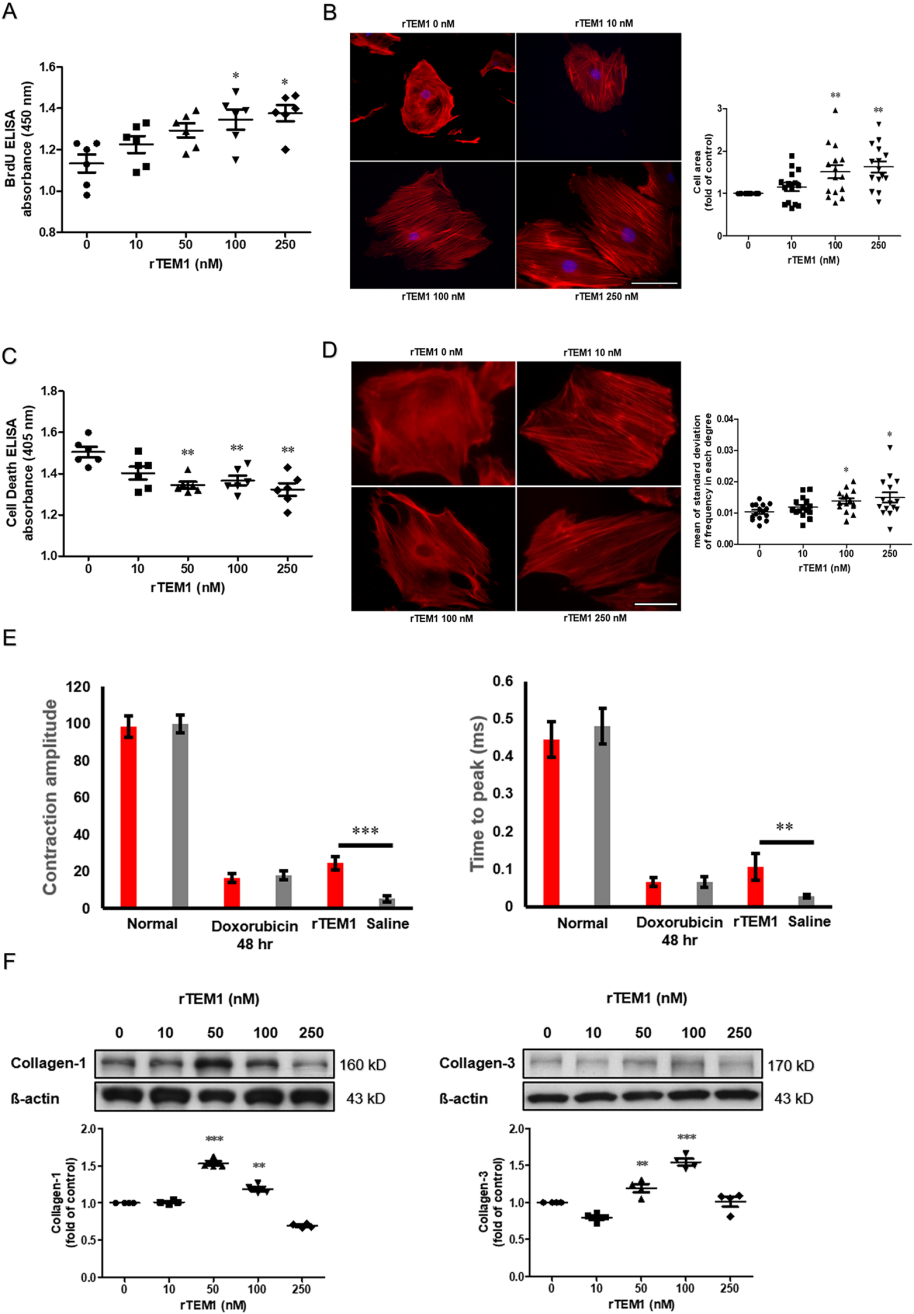
TEM1 affected cell behaviors (**A**) Cell proliferation was evaluated by measuring BrdU incorporation with ELISA during DNA synthesis as the cells replicate (n = 6). The data were presented as absolute values of absorbance. *p < 0.05 compared to control group adding saline. (**B**) Cell area was measured and expressed as a ratio of rTEM1-to saline-treated cells (15 cells were measured after each treatment). **p < 0.01 compared to control group with saline treatment. Scale bar = 100 mm. (**C**) Cell apoptosis was measured by quantifying oligonucleosomes cleaved from double-stranded DNA with ELISA during cell apoptosis (n = 6). The data were presented as absolute values of absorbance. *p < 0.05; **p < 0.01 compared to control group adding saline. (**D**) Representative pictures showed more uniform orientation of actin filaments after rTEM1 treatment (15 cells were measured after each treatment). Scale bar = 100 mm. Images of actin filament were analyzed by Fast Fourier Transform and standard deviations of actin filament direction were calculated. *p < 0.05 between comparisons. (**E**) Contraction amplitude and velocity of the hESC-derived contractile cardiomyocytes were measured after indicated treatment (n = 4). **p < 0.01 and ***p < 0.001 compared to control group with adding saline. (**F**) Collagen-1 and collagen-3 were evaluated with western blot. The expression levels were expressed as a ratio to normal without adding rTEM1 (n = 4). **p < 0.01 and ***p < 0.001 compared to normal. Original images of western blot were provided in “Supplemental file”.

Treatment of H9C2 cells with rTEM1 increased cell proliferation (Fig. 4A), induced cell hypertrophy (Fig. 4B) and decreased doxorubicin-induced cell apoptosis (Fig. 4C). The assembly of actin filaments into aligned structures governs cell shape, movement and contractility in muscle cells. We found rTEM1 treatment caused more organized alignment of actin filaments (Fig. 4D). To verify the influence of rTEM1 on cell contractility, hESC–derived contractile cardiomyocytes were developed. rTEM1 treatment could partially rescue the impaired contractility in doxorubicin-treated cardiomyocytes (Fig. 4E). Treatment of H9C2 cells with rTEM1 increased collagen production (Fig. 4F). However, collagen production was decreased with high dose rTEM1. The possible explanations are that H9C2 cells had expressed maximal capacity of collagen production under lower dose of rTEM1 stimulation or very high concentration of TEM1 had negative impact on collagen secretion in cardiomyocytes.

### Cardiac function in TEM1^lacZ/lacZ^ mice

**Figure 5:**
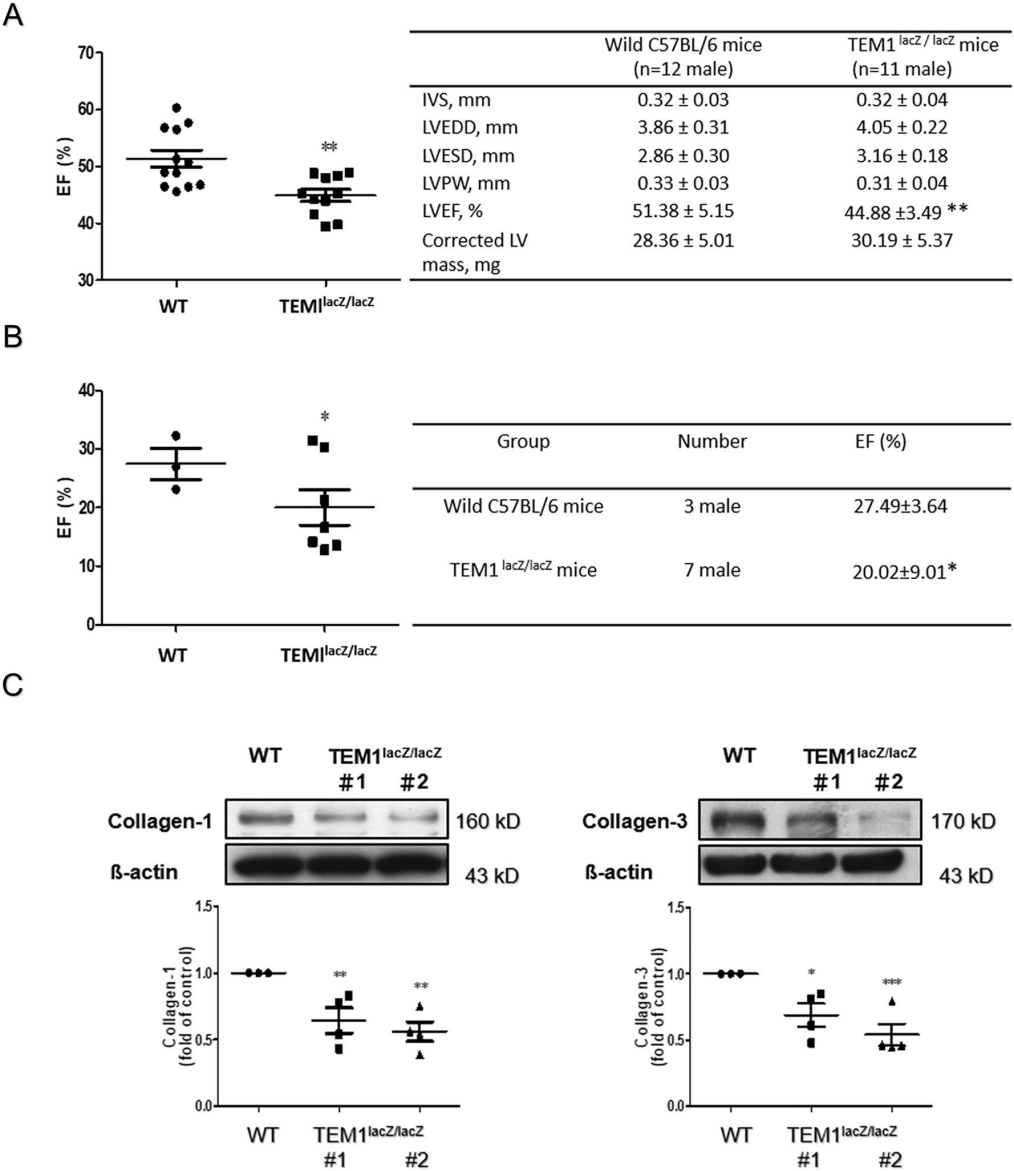
Cardiac contractility and collagen content of TEM1^LacZ/LacZ^ mice. (**A**) Cardiac contractility evaluated by echocardiography. *p < 0.05 compared to wild type mice. IVS, interventricular septum; LVEDD, left ventricular end-diastolic dimension; LVESD, left ventricular end-systolic dimension; LVPW, left ventricular posterior wall; LVEF, left ventricular ejection fraction. (**B**) Cardiac contractility evaluated by pressure volume loop analysis. **p < 0.01 compared to wild type mice. (**C**) Collagen expression levels in 2 hearts of TEM1^LacZ/LacZ^ mice (#1 and #2) were analyzed by western blot (n = 4). *p < 0.05 compared to wild type mice. Original images of western blot were provided in “Supplemental file”.

Compared with the wild type, the LV systolic function was slightly lower in TEM1^lacZ/lacZ^ mice evaluated by echocardiography (Fig. 5A) and pressure volume loop analysis (Fig. 5B). There were less collagen contents, including collagen type 1 and 3 detected from the excised hearts of TEM1^lacZ/lacZ^ mice (Fig. 5C).

### Signaling pathways

**Figure 6:**
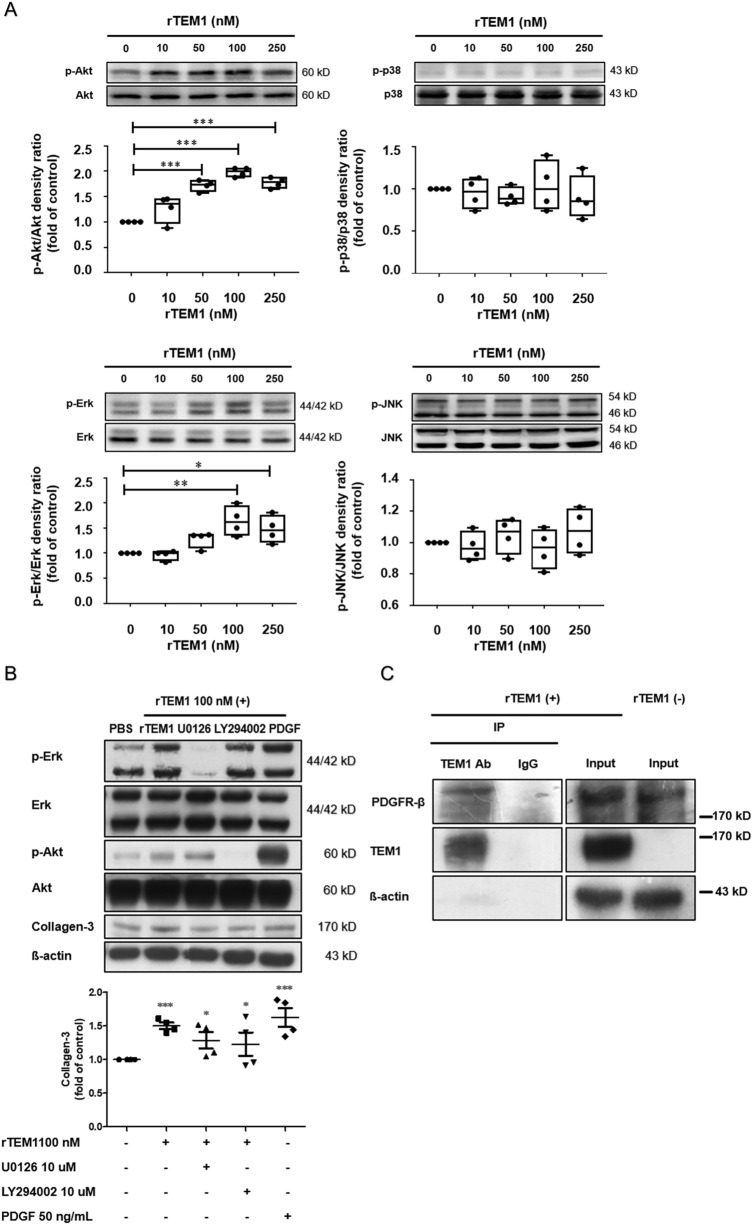
Signaling pathways. (**A**) Western blot of p-Akt, p-ERK, p38 and p-JNK in H9C2 cells after rTEM1 treatment (n = 4). *p < 0.05, **p < 0.01 and ***p < 0.001 compared to control without adding rTEM1. (**B**) rTEM1-induced collagen production in H9C2 cells was reduced after adding U0126 (Erk inhibitor) or LY294002 (Akt/PI3 kinase inhibitor) (n = 4). PDGF was used as a positive control. **p < 0.01 compared to rTEM1 or PDGF treatment and ***p < 0.001 compared to PBS treatment. (**C**) Coimmunoprecipitation of TEM1 and PDGF receptor with or without rTEM1 treatment. H9C2 cells were immunoprecipitated with anti-TEM1 antibody or control IgG (n = 4). Pellets were then subjected to western blot with anti-TEM1 or anti-PDGF receptor antibodies. Original images of western blot were provided in “Supplemental file”.

Finally, we looked at the potential signaling cascades of TEM1 in cardiomyocytes. Phosphorylation of 4 signaling proteins was evaluated. The Akt/PKB (protein kinase B) kinase and extracellular signal-regulated kinase (Erk) pathways were activated in the H9C2 cells after rTEM1 treatment (Fig. 6A). There were no changes of the c-Jun amino-terminal kinase (JNK) and p38 mitogen-activated protein kinase pathways. Akt or Erk inhibitor treatment could partially suppress rTEM1-induced collagen production in H9C2 cells (Fig. 6B). Coimmunoprecipitation showed that after rTEM1 was pulled down by anti-TEM1 antibody, platelet-derived growth factor (PDGF) receptor signal can be observed in H9C2 cell lysates (Fig. 6C). These results indicated that TEM1 is involved in the binding of PDGF receptor and activation of PDGF downstream signaling pathway in cardiomyocytes.

## Discussion

This study found TEM1 expression in cardiac specimens taken from patients with heart failure and mouse heart failure models. Cardiac injury induced TEM1 expression in the cultured cardiomyocytes. Moreover, rTEM1 affected cardiomyocyte behaviors changes that participate in remodeling after cardiac injury and maintaining normal cardiac function.

In current study, we first found rTEM1 changed the cell behaviors of cardiomyocytes, including hypertrophy, hyperplasia, apoptosis and collagen production, all of which are essential adaption responses during the process of cardiac remodeling^13,14^. Previous studies have found that re-expression of TEM1 in activated cells of the mesenchymal lineage during pathological conditions in other tissues. TEM1 is highly expressed in tumor vessel-associated perivascular cells (pericytes), myofibroblasts, stroma cells and increases cancer cell migration and angiogenesis^23,24^. In patients with colorectal cancer, soluble TEM1 fragments with molecular weight ranged from 120 to150 KDa can be found in the systemic circulation^25^. In atherosclerosis, there is TEM1 expression in vascular smooth muscle cells (VSMC). TEM1 changes the cell behaviors of VSMC by shifting VSMC toward a proliferative and synthetic phenotype^11^. In response to hepatic injury, TEM1 expression is significantly increased in hepatic stellate cells and myofibroblasts in the perisinusoidal space of liver. Deletion of TEM1 demonstrated decreased proliferative response of hepatic stellate cells to PDGF stimulation and also has less collagen production^10^. The results from these studies and our study indicated that expression of TEM1 altered mesenchymal lineage-derived cell behaviors. TEM1 is a functional protein that responses to injury and involves in the pathophysiological process of organ damage including the heart.

Actin filament is one of the fundamental filamentous proteins which consist of cytoskeleton in various cell types and responsible for maintenance of cellular shape, and processes of cell dividing, adhesion, and motility^26^. There are several types of arrays of actin organized within cells, such as parallel bundles, gel-like or dendritic weblike networks. Our study observed that rTEM1 induced actin filaments to form more parallel bundles array in a dose-dependent fashion in H9C2 cells. In contractile muscular cells, such as cardiomyocytes, highly parallel organized actin filaments are necessary to cooperate with myosin filaments and other regulatory proteins to form the essential unit of contraction unit. This result made us to further look at the direct influence of rTEM1 on cell contractility and demonstrated that rTEM1 could directly rescue the contractility of doxorubicin-damaged cardiomyocytes. However, the detailed molecular mechanisms responsible for the rTEM1-induced actin rearrangement are not clear and needs further studies. Collagen is the predominant constituent of ECM in the heart wherein collagen 1 and 3 account for the majority of structural framework of the healthy cardiac ECM^27,28^. Our study found that TEM1-deficient (TEM1^lacZ/lacZ^ mice) mice had less collagen contents than wild-type mice. In renal fibrosis, stromal fibroblasts expressing TEM1 are the primary cells that produce fibrotic matrix. Expression of TEM1 is associated with the extent of fibrosis in patients with IgA nephropathy and TEM1 knockout mice have less renal fibrosis^9^. Moreover, TEM1 also influenced collagen deposition and affected wound healing^12^. Our finding implied that TEM1 might play a role in ECM formation and collagen deposition in the development of normal healthy cardiac tissue. ECM and collagen in the heart provide architectural support, involving in maintaining ventricular geometry, facilitating myocyte-generated force transmission, and transducing signals required for contractile function^27,28^. It is likely that that the less collagen deposition in the heart of TEM1-deficient mice may also contribute to the worse cardiac contractile function we observed in our study. Altered collagen composition is associated with changes in ventricular stiffness and function^29^. Recent studies showed that restoration of collagen might prevent adverse cardiac remodeling, and improve impaired systolic function in the heart of animal ischemia/infarction models^30,31^. Based on these study results and our findings, TEM1 might be a potential therapeutic target in treating heart failure by improving cardiomyocyte contractility and renovating collagen content in the damaged heart after cardiac injury.

In response to stress stimuli, the mitogen-activated protein kinase (MAPK) family of proteins, including Erk, p38, and JNK, mediate signal transduction from the cell surface to the nucleus and influence changes of multiple cell behaviors. We found TEM1 directly increased the expression levels of phosphorylated Erk and protein kinase B (Akt) in the H9C2 cells. Previous studies have shown that TEM1 directly regulated the PDGF-induced activation of Erk in fibroblasts and Erk is not phosphorylated in response to PDGF if there is no TEM1 expression^10,12^. There was also a close colocalization of TEM1 and PDGF receptor in fibroblasts^12^. PDGF-induced cell proliferation was abolished in hepatic stellate cells from TEM1^−/−^ mice^10^. PDGF receptor signaling in cardiomyocyte becomes quiescent after birth, but the expression and activation of PDGF receptor rise dramatically in response to cardiac stress and changes the cell behaviors of cardiomyocytes^32,33^. We found there was TEM1 binding to PDGF receptor in H9C2 cells. TEM1 could directly interact with the PDGF receptor in cardiomyocytes and participate the downstream signal transduction and changes of cell behaviors. Cardiac remodeling and heart failure involve the distinct processes of cardiomyocyte hypertrophy, alteration of extracellular matrix, fibrosis, inflammation and many other complex mechanisms. Current study proves that TEM1 may participate in some of these mechanisms. More studies are needed to explore how TEM1 affects cell behavior changes not only in cardiomyocytes but also in cardiac fibroblasts and how these changes may influence cardiac remodeling and heart failure.

In conclusion, the present in vitro and in vivo studies showed that cardiac injuries stimulated TEM1 expression in cardiomyocytes. Moreover, rTEM1 affected cellular behaviors of cardiomyocytes which participate in the processes of cardiac remodeling. TEM1-deficient mice had less collagen content and worse cardiac function than wild mice indicating that TEM1 plays a role in maintaining normal cardiac structure and function.

## Supplementary Information

Supplementary Figures.
