# Supplementary figures and images for: Tumor endothelial marker 1 is upregulated in heart after cardiac injury and participates in cardiac remodeling
Source: Sci Rep. 2022 Jun 22;12:10532. doi: 10.1038/s41598-022-14567-2 (PMC9218118; doi:10.1038/s41598-022-14567-2)

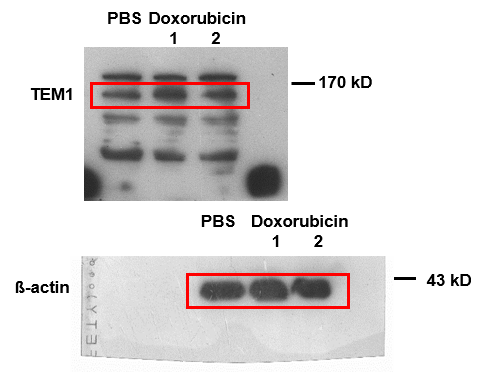

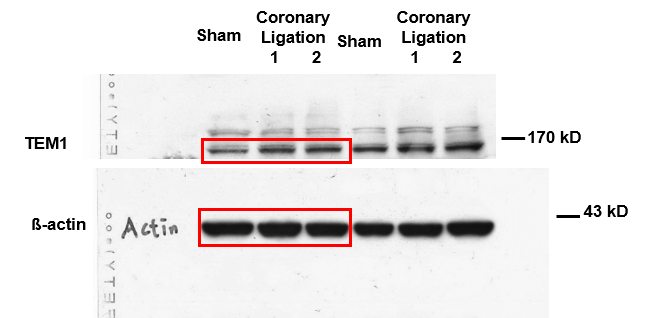


E

Figure 2

Figure 3


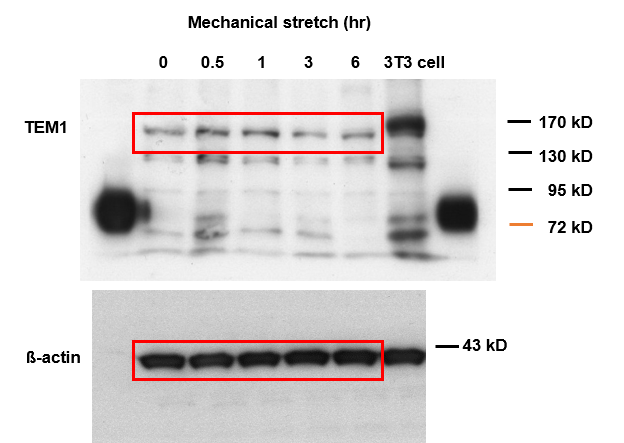


A

B


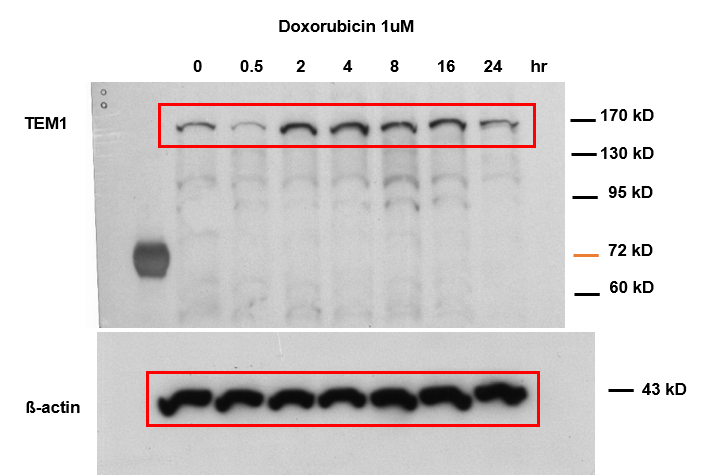


C


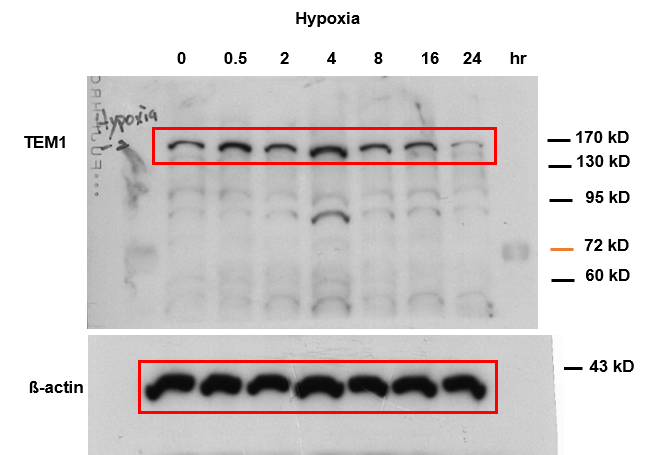


F

Figure 4


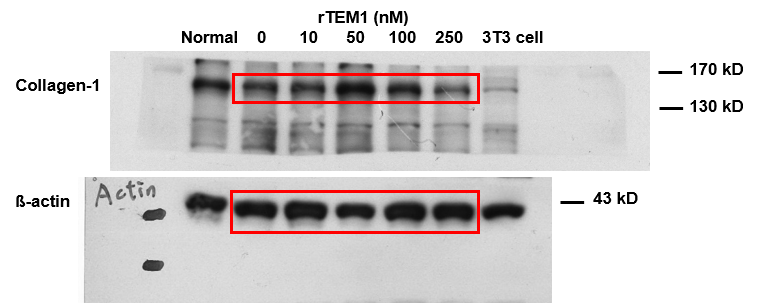


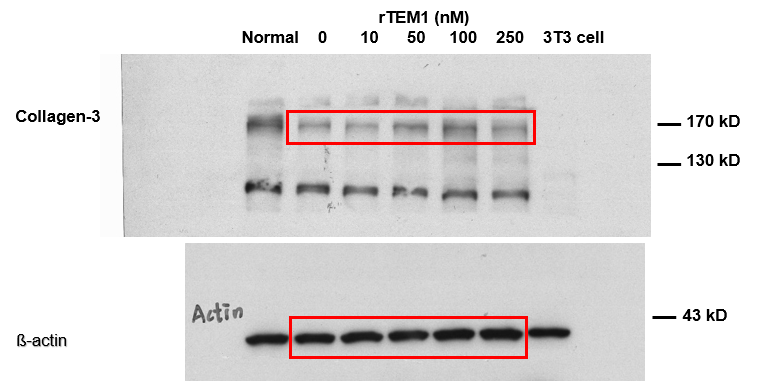


Figure 5

C


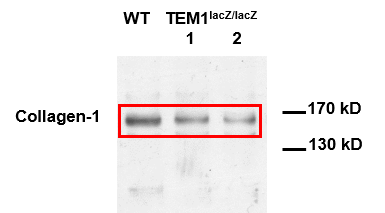

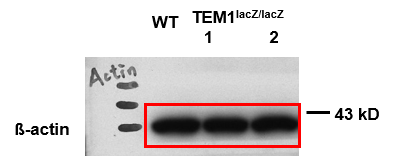

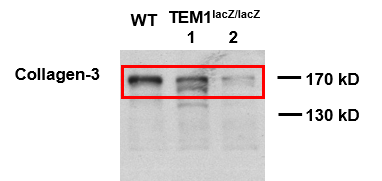

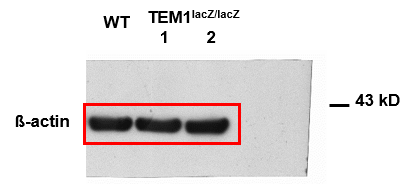


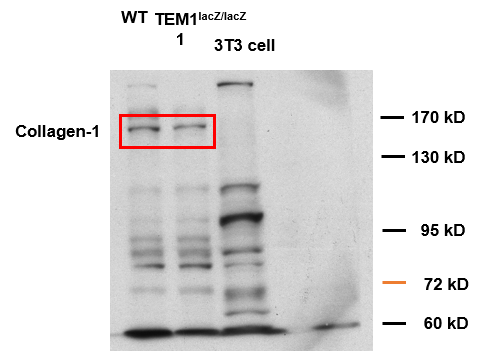

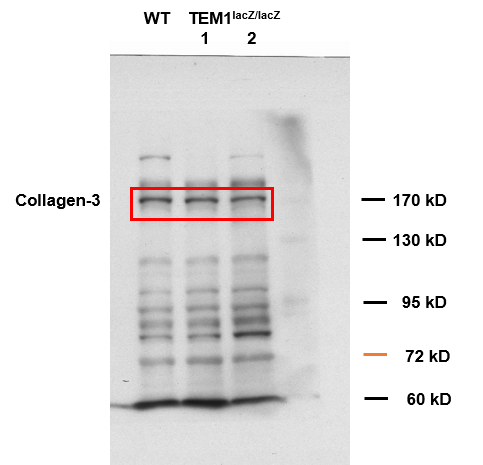


A

Figure 6


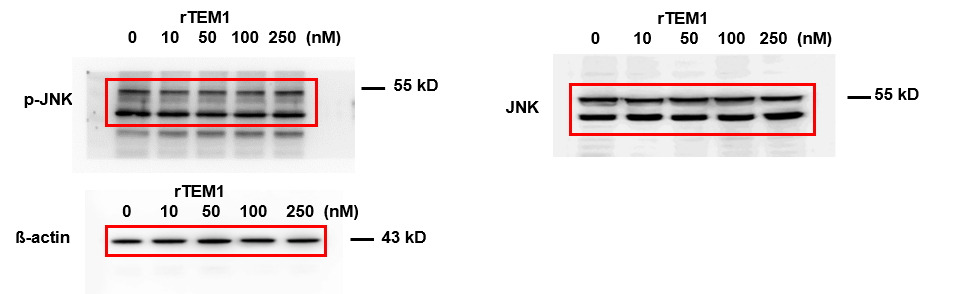

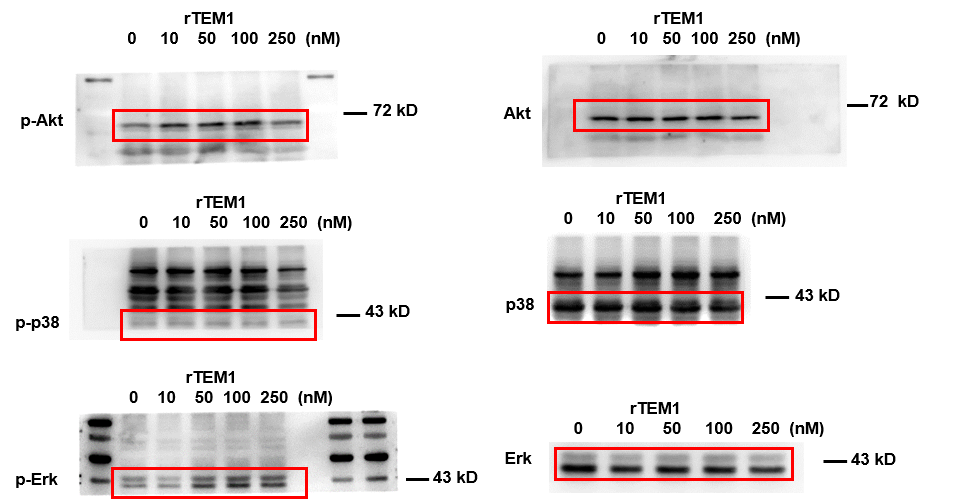


B


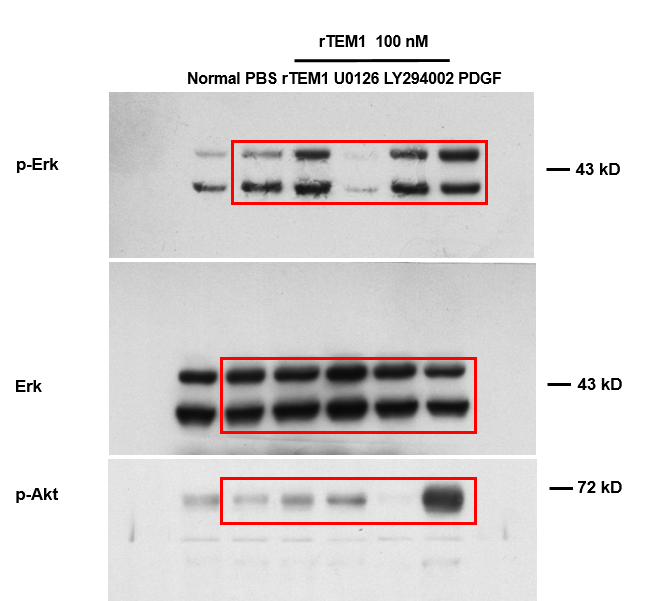

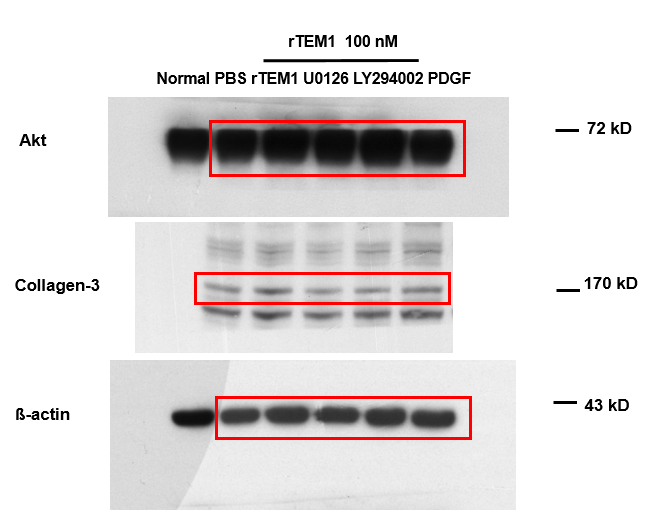


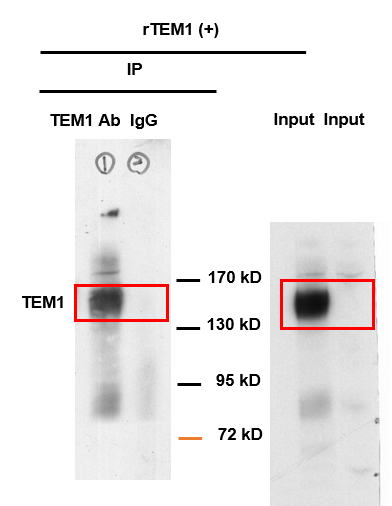

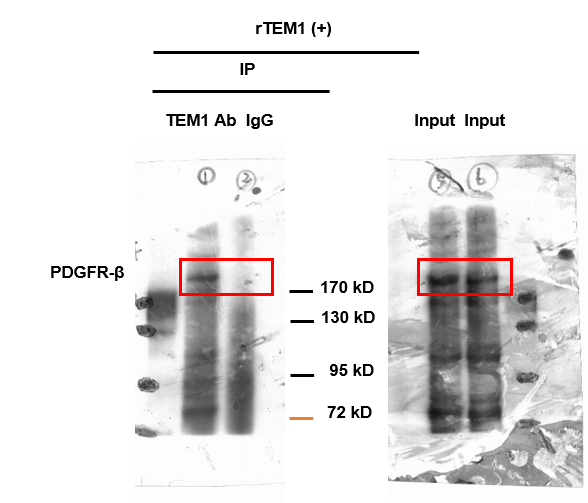


C


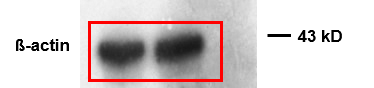

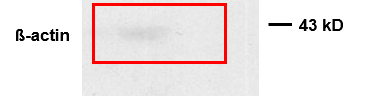

Supplement: Supplementary file 1 — Supplementary Figures. [file 41598_2022_14567_MOESM1_ESM.docx]
